# Low cost Ti–Si intermetallic compound membrane with nano-pores synthesized by in-situ reactive sintering process

**DOI:** 10.1038/s41598-020-73869-5

**Published:** 2020-10-07

**Authors:** Zhongjun Liu, Zhuomeng Liu, Shuai Ji, Gaosong Wang

**Affiliations:** 1 School of Materials Science and Engineering, Xi’an Shiyou University, Xi’an, 710065 Shaanxi China; 2 Key Laboratory of Electromagnetic Processing of Materials, Ministry of Education, Northeastern University, Shenyang, 110819 China

**Keywords:** Materials science, Nanoscale materials

## Abstract

A low cost Ti–Si intermetallic compound membrane with nano-pores was successfully prepared by an in-situ reactive sintering process. The all synthesized membrane shows the presence of Ti, Ti_5_Si_3_, TiSi and TiO_2_ phases, and the Ti:Si atomic ratio of membrane is about 1.9. Two different synthesized granule configuration zones on membrane are observed. Membrane synthesized on the surface of Ti particles contains the mean sizes of both 631 nm nano-particles and 238 nm nano-pores, which is considerably different from that of membrane growing on top of the micro-pores of Ti matrix, 238 nm nano-particles and 80 nm nano-pores, respectively.

## Introduction

Porous membrane can be fabricated from a wide variety of materials, and typically classified as organics^1,2^, inorganics^3,4^ and organic–inorganic composites^5^. Since most organic porous membrane can not endure the conditions of high temperature and organic solvents, traditional inorganic porous membranes, such as porous metal and porous ceramic, are attracted most attentions and increasing interest in the fields of filters, heat exchangers, solid oxide fuel cell electrodes, and biological materials etc^6–10^. Especially, basing on the characteristics of good machinability, high strength at ambient temperatures, and the enough impact energy absorption capacity, metal porous membrane has been wildly used in some special industrial fields.

Usually pore size and structure are tailored to best serve the application required, and asymmetric pores is one of the most common structure for porous membranes^11^. Asymmetric porous membrane is the combination of multiple layers with large pore support layer, intermediate layer/transition layer, and small pore active layer. Although the intermediate layers with gradients in pore size are frequently used to connect the support and active layers, it partly decreases the overall permeability. Theoretically, if one can directly construct the active layer on support layer perfectly, asymmetric porous membrane should possess a better permeability and selectivity performance.

For example, porous metal membranes with pore size gradient structure having high filtering accuracy and high flux are expected for filtration application. As porous support layer can be metals and ceramics, the fabrication technology of active layer vary considerably and often extend to the coating process, involving PVD, CVD, electrochemical, dip and sol gel process, etc^3,12^. After a sophisticated fabrication process, the active layer with ether micro-pores or nano-pores can be obtained on metal support layer. Nevertheless, the complexity of these coating process limits the manufacture for coating on a large piece of porous metal and forms a uniform nanopore structure.

Metal silicides, as a representative materials for high temperature environments, have received considerable attentions in advanced areas^13–18^. Various metallurgical processes are developed to synthesize Ti-Si intermetallic compound, such as arc-casting, cold and hot pressing, thermal spraying, reactive sintering^19–21^. Our previous work has proved the method practicability of in-situ reactive sintering in in confined space^22^, to synthesize membrane with nanopores; and revealed that the phase diffusion reaction between SiO_2_ and Ti is accompanied by the formation of intermediate phases such as Ti_5_Si_3_ during the in-situ reactive sintering process. In this work, the nano-pore structure characteristics of porous Ti–Si membranes are studied, and the distributions of synthesized Ti–Si particles size and pore size of membrane are demonstrated.

## Experimental

### Methods

**Figure 1:**
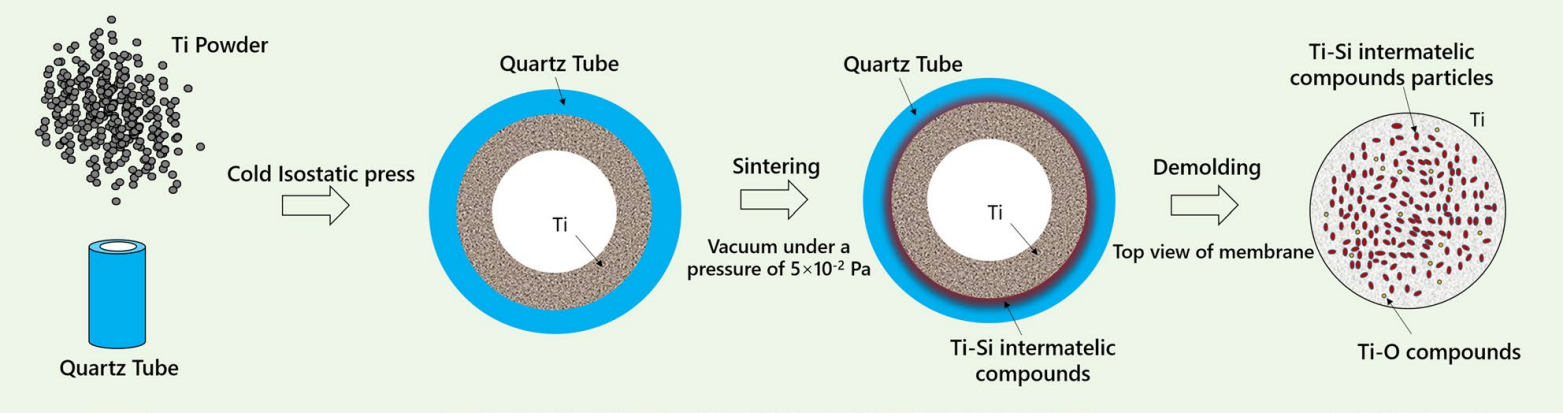
Schematic illustrations showing the fabrication of Ti–Si intermetallic compound porous membrane by in-situ reactive sintering process.

In our present work, a novel, but simple method of in-situ reactive sintering in confined space is reported for producing Ti–Si intermetallic compound porous membrane for the first time^22^. The strategy of this method is schematically shown in Fig. 1. Firstly, Ti powder was cold-isostatic-pressed onto the inner surface of quartz tube, and then porous titanium on the inner wall of quartz tube were sintered in a vacuum condition. Finally, the Ti–Si porous membrane was synthesized on the interface between the outer surface of porous Ti substrate and the inner surface of quartz tube.

### Fabrication

Two Ti powders (99.9 wt.%) were obtained by sieving, coarse powder (47 < size < 75 µm, D_50_ = 56.8 µm) and fine powder (size < 47 µm, D_50_ = 23.4 µm). Ti powders was firstly cold-isostatic-pressed onto the inner surface of quartz tube (SiO_2_ ≥ 99.5 wt.%) under pressing pressure of 120 MPa. The inner diameter of the quartz tube is 15 mm with 2.5 mm in thickness, and the porous titanium matrix performs with dimension of ⌀ 15 mm in outer diameter and 1.2 mm in thickness. Porous Ti matrix with quartz tube was sintered at a vacuum atmosphere (5 × 10^–2^ Pa). The sintering condition of fine powder was 960 ∘C for 120 min, and the coarse powder was 1020 ∘C for 120 min and 60 min, respectively. The rate of temperature increasing and decreasing were 3 ∘C/min and cooling with furnace.

### Characterization

Thermal expansion coefficient tester (EXSTAR6000) was used to see the sintering expansion difference between porous matrix and quartz tube. The morphology of Ti-Si porous membrane was observed using Scanning Electron Microscope (SEM, JSM-6390A), and the acceleration voltage during EDS measurement is 15 kV. X-ray diffraction (XRD) scans were acquired with the Shimadzu XRD-6000 and Bruker D8 Advance systems, respectively. The surface composition and elemental chemical state of the membranes were examined by X-ray photoelectron spectroscopy (XPS) using a Model Axis Ultra DLD (SHIMADZU) apparatus.

## Result and discussion

**Figure 2:**
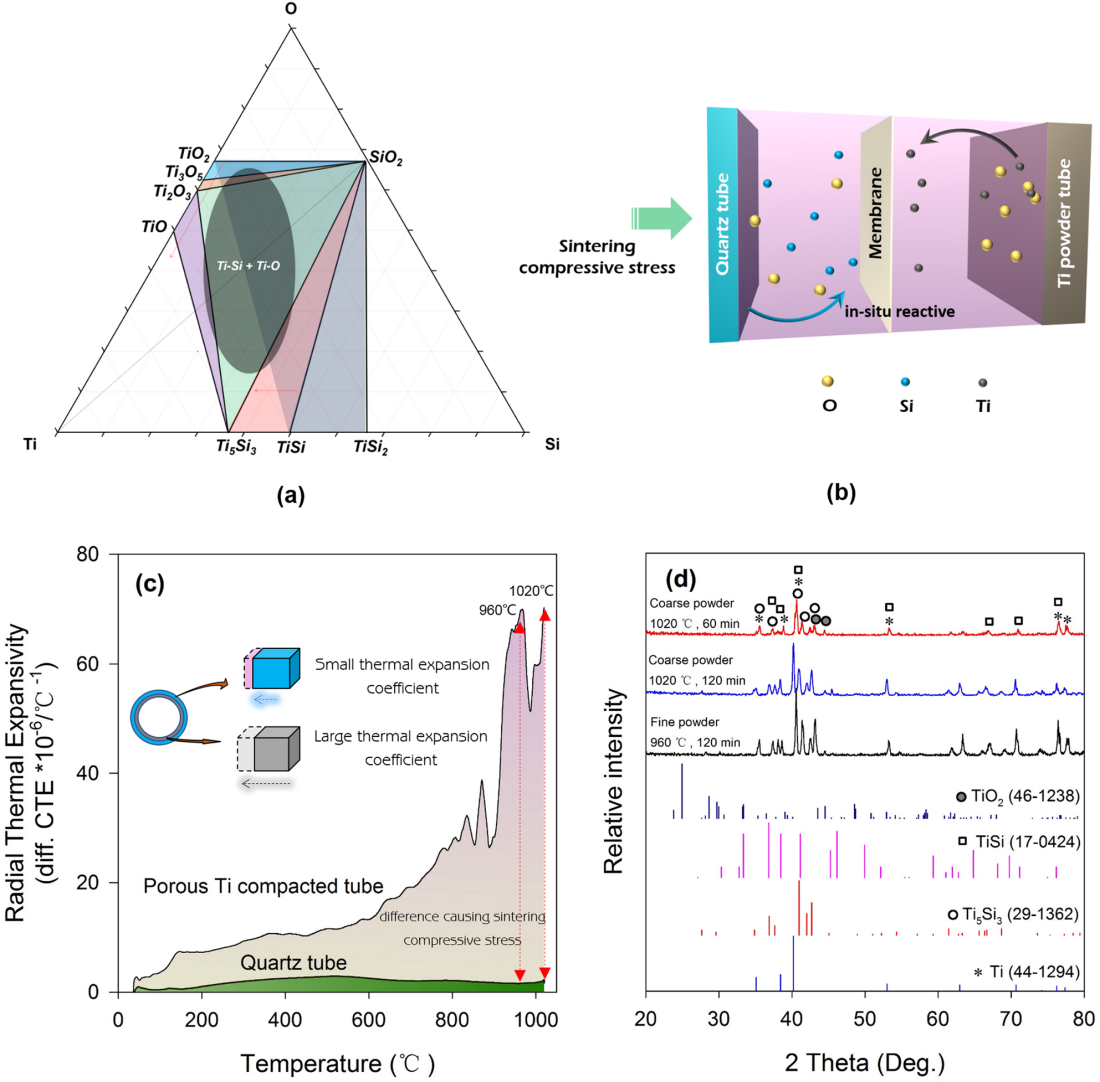
**(a)** Ti–Si–O ternary phase diagram, **(b)** the schematic illustration of Ti–Si intermetallic compound membrane synthesis process, **(c)** the radial thermal expansivity of porous Ti matrix and quartz tube, **(d)** the X-ray diffraction (XRD) patterns of Ti–Si porous membrane.

The idea of in-situ reactive sintering process in confined space is basing on the composition paths of the various diffusion couples in the ternary phase diagram^23,24^, as shown in Fig. 2a. Apparently, quartz (SiO_2_) is the best choice to provide Si and O, considering the effects of economic factors. Figure 2b shows the schematic illustration of Ti–Si intermetallic compound membrane synthesis process. During the in-situ reactive process between SiO_2_ and Ti, since the diffusion of O in Ti is fast compared to the SiO_2_ decomposition reaction, the SiO_2_ should be decomposed under the influence of Ti, and the oxygen diffuses rapidly toward to Ti particles^25,26^.

Figure 2c shows the radial thermal expansivity of porous Ti matrix and quartz tube. Obviously, there is a large difference during sintering process, and a lager thermal expansion coefficient of porous Ti results in a compressive stress on the quartz tube. This sintering compressive stress is beneficial to in-situ reactive and the formation of Ti-Si porous membrane. It is analogue to a pressure-sintering process. Figure 2d shows the X-ray diffraction (XRD) patterns of Ti–Si porous membrane. The all synthesized membranes show the presence of Ti, Ti_5_Si_3_, TiSi and TiO_2_ phases. This indicates that the whole reactive synthesis can be expressed as simple reaction equations of 3SiO_2_ + 8Ti → Ti_5_Si_3_ + 3 TiO_2_, and SiO_2_ + 2Ti → TiSi + TiO_2_. As SiO_2_ decomposed quickly with heating, O diffused fast toward to Ti matrix^24^. Then in the case of Ti-Si, the phase formation has been attributed to the immobility of Ti in Si at the interdiffusion temperature. The phase of only Ti_5_Si_3_ and TiSi obtained is because of the relative low free energy during formation at in-situ reactions^23^. For the membrane fabricated by coarse powder, the amount of Ti_5_Si_3_ increased as extending the holding time. In terms of the fine powder, it was observed more Ti–Si than that of coarse powder in this work.

**Figure 3:**
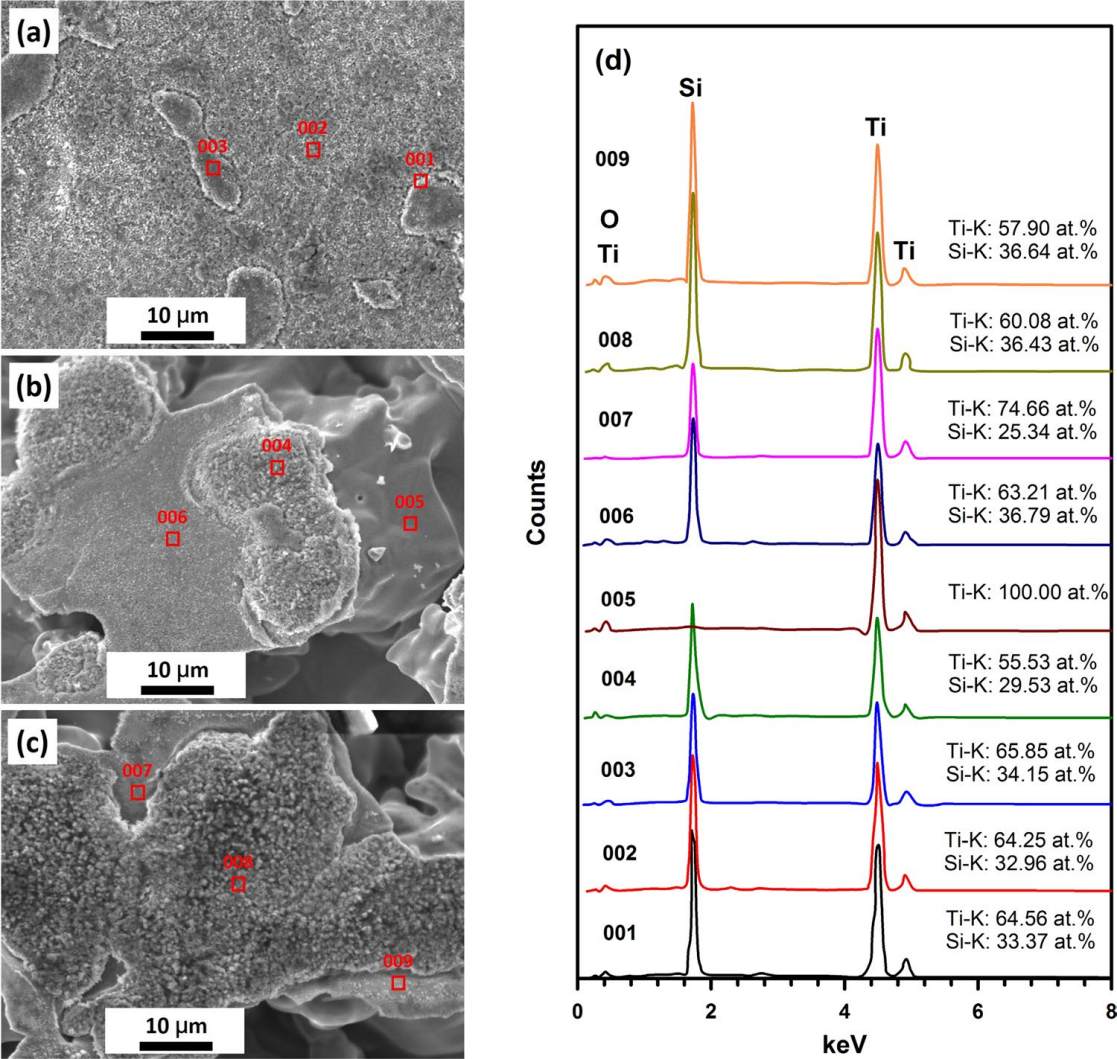
The EDS data of porous membrane fabricated by fine and coarse powder: **(a)** fine powder, 960 ℃, 120 min; **(b)** coarse powder, 1020 ℃, 120 min; **(c)** coarse powder, 1020 ℃, 60 min; **(d)** EDS data of point 001 to 009.

EDS point measurements were performed to further determine the elemental distributions (Fig. 3d). The detected points chosen for EDS are the areas of porous membrane on top of Ti particles and pores. For the membrane synthesized by fine powder (Fig. 3a), Ti and Si elements were detected with the Ti:Si atomic ratio of about 1.9. Here it should be noted that there is no O element detected at point 003. At the beginning of in-situ reaction, SiO_2_ is decomposed under the influence of Ti, and the oxygen is distributed rapidly over the Ti matrix, due to the diffusion of O in Ti is fast compared to the SiO_2_ decomposition reaction^24^. With the process of reaction, the solubility limit of oxygen in Ti is exceeded and the formation of a titanium oxide occurs. This is the reason of O element ununiformly distributed and is not observed at some points. Similarly phenomena are obtained for Fig. 3b,c. One thing should be mentioned that only Ti element is detected for Ti particle surface of matrix (Fig. 3b, point 005).

**Figure 4:**
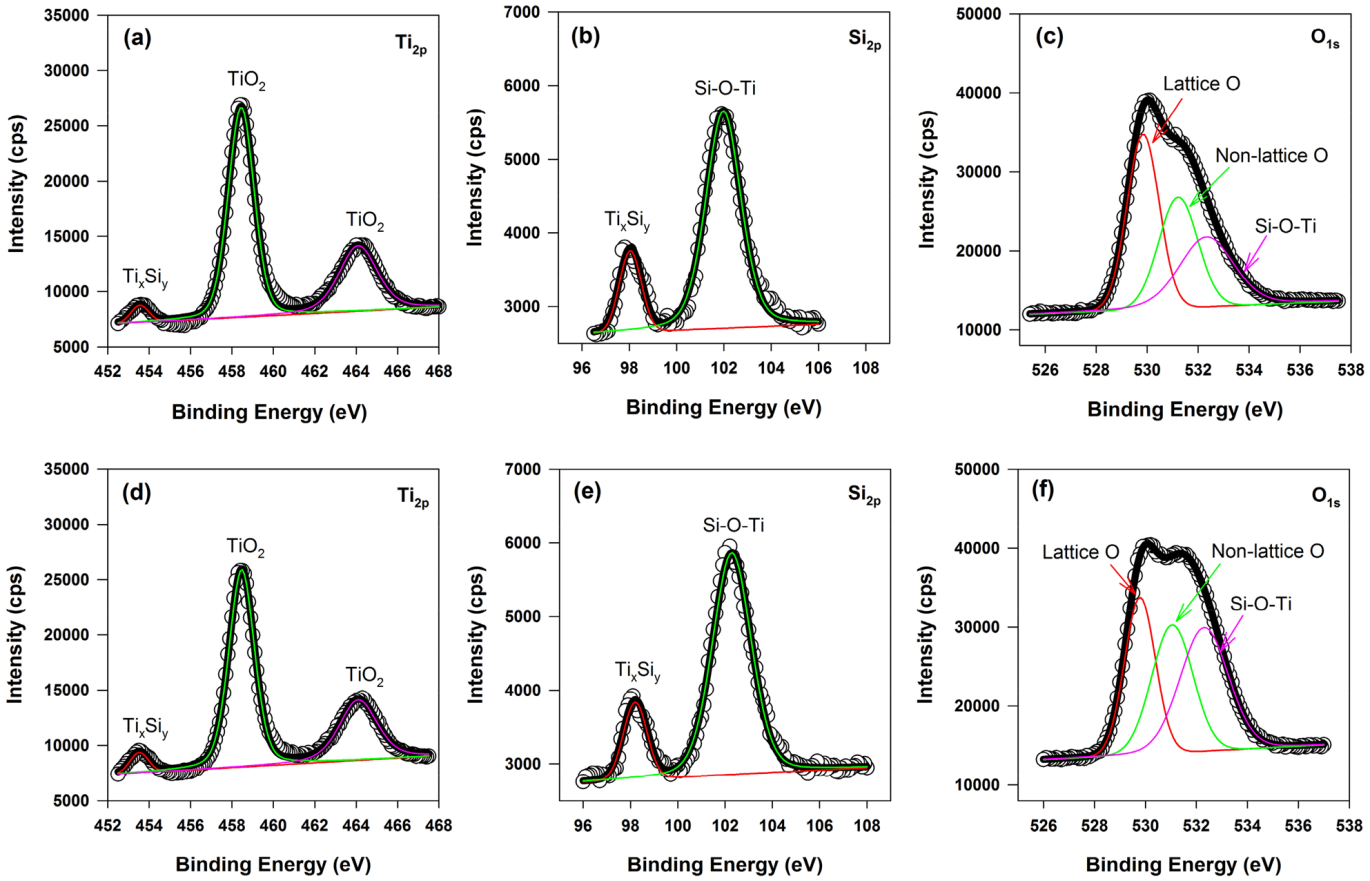
High resolution XPS spectra of Ti 2p, Si 2p and O 1s in the membranes: **(a–c)** coarse powder, 1020 ℃, 120 min; **(d–f)** fine powder, 960 ℃, 120 min.

In order to further characterize the composition states of the titanium, silicon and oxygen ions, XPS spectra of Ti–Si membranes were obtained. Shown in Fig. 4a and (d), the binding energies of Ti 2p appearing at 453.5 eV indicate the presence of titanium silicide (Ti_x_Si_y_)^27–29^; the peaks appearing at 458.6 eV and 464.4 eV correspond to the membrane of Ti 2p of TiO_2_^27,30^. The binding energy of the Si 2p appearing at 98.2 eV reconfirmed the formation of Ti_x_Si_y_^31^; also the binding energy peaks of 102 eV (Fig. 4b) and 102.3 eV are close and the same to the reported values (e.g. 102.3 eV^32,33^) for that of Si–O–Ti bonds. This implies that Si of decomposed SiO_2_ is forced to enter the crystal lattice of TiO_2_ as interstitial atoms to establish the Si–O–Ti bond during the synthesis process^34^. Next, the O 1s spectrum of membrane are shown in Fig. 4c,f, which are fitted with three peaks. The peaks at binding energies 529.8 eV, 531.2 eV and 532.3 eV are attributed to lattice oxygen, non-lattice oxygen and Si–O–Ti^30,32^. The peak value of binding energy of O 1s at 532.3 eV reconfirmed the formation of Si–O–Ti bonds as the results obtained in Fig. 4b,e).

**Figure 5:**
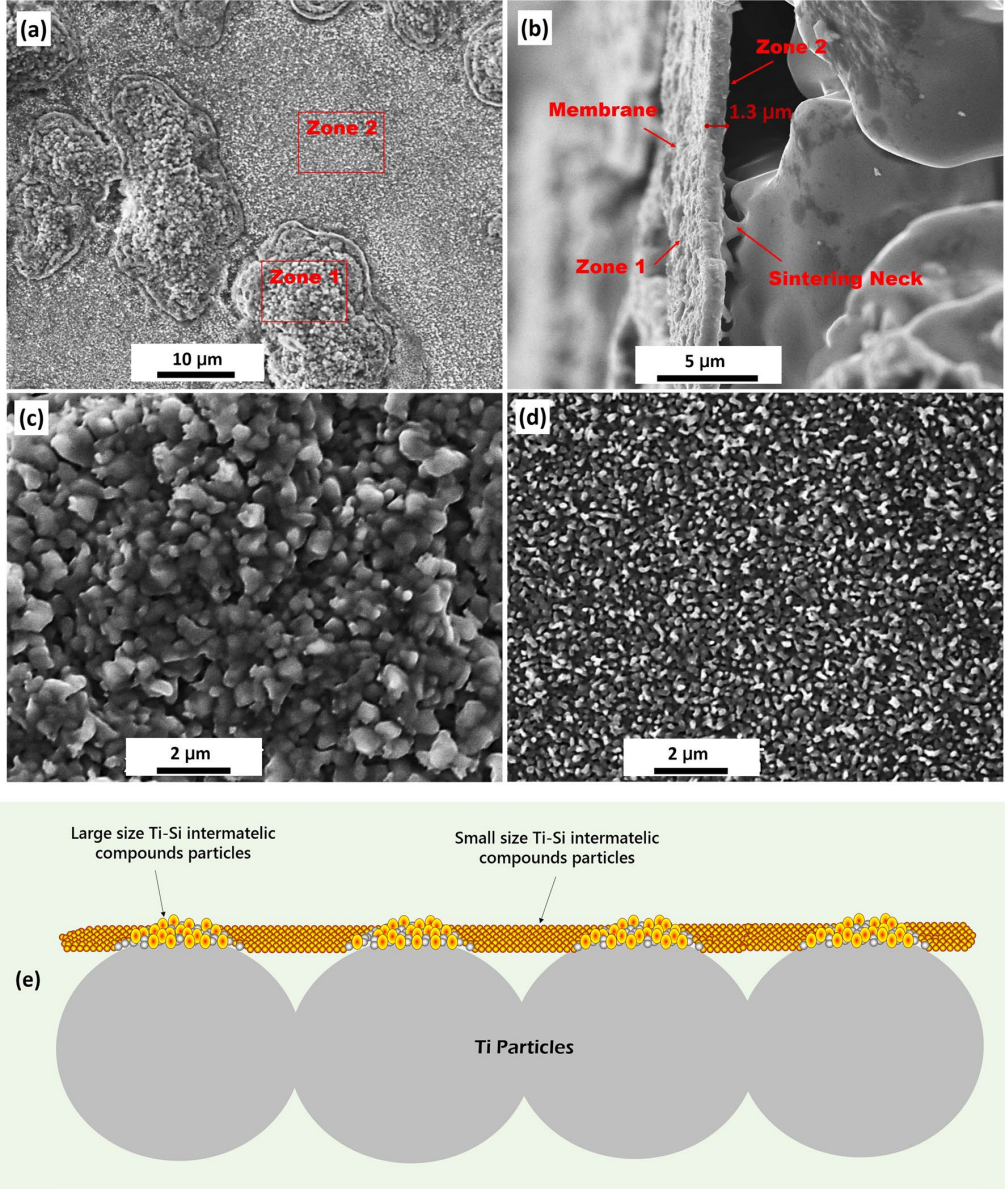
The SEM images of membrane prepared by coarse powder (1020 ℃, 60 min): **(a)** surface; **(b)** cross section. **(c**, **d)** are the SEM images with higher magnification corresponding to zone 1 and 2 indicated in **(a)**, respectively; **(e)** is the schematic illustration of different microstructures of membrane.

SEM images of the membrane surface of Ti–Si intermetallic compound buildup on porous Ti matrix prepared by coarse powders (1020 ∘C, 60 min) are shown in Fig. 5. Figure 5a shows two different synthesized granule configuration zones, which can be seen clearly in Fig. 5c,d, respectively. For zone 1 (Fig. 5c), it is indicated a uniform particle distribution, and the particle size is larger and more closely arranged compared with zone 2 (Fig. 5d). Figure 5e schematically illustrates the different microstructures of membrane. During in-situ reaction, Ti particles contact with quartz tube (SiO_2_) surface (Zone 1), the distance between Ti and SiO_2_ is shorter, and it is beneficial to the inter-diffusion and reaction. This results in zone 1 as an over-reaction area. On the contrary, zone 2 is where Ti particles did not contact with quartz tube, and the formation of porous membrane is attributed to the longer distance inter-diffusion of Ti and SiO_2_.

Figure 5b shows the longitudinal section SEM image of the porous membrane. It can be clearly seen that the membrane presents a sidestep microstructure with the thickness of about 1.3 µm. Sidestep microstructure in the longitudinal section indicates the presence of open pores throughout the membrane. In our present work^22^, it has been shown that the membrane thickness prefers to be constant (1–3 µm). Reasons for this are twofold. First, the sintering stress, resulted from the difference of thermal expansion coefficient between porous Ti matrix and silica, restricts the thickness growth of the membrane. Secondly, the volume increment during the in-situ transformation of titanium and silica into Ti–Si intermetallic compound phase hinder the thickness growth. Since membrane of zone 1 is overgrowth on the surface of Ti particle, the effects of more closely arranged particles can be ignored on the filtration accuracy and flux. In addition, sintering neck between membrane and Ti matrix was observed, indicating a good metallurgy bonding and an effective sintering process.

**Figure 6:**
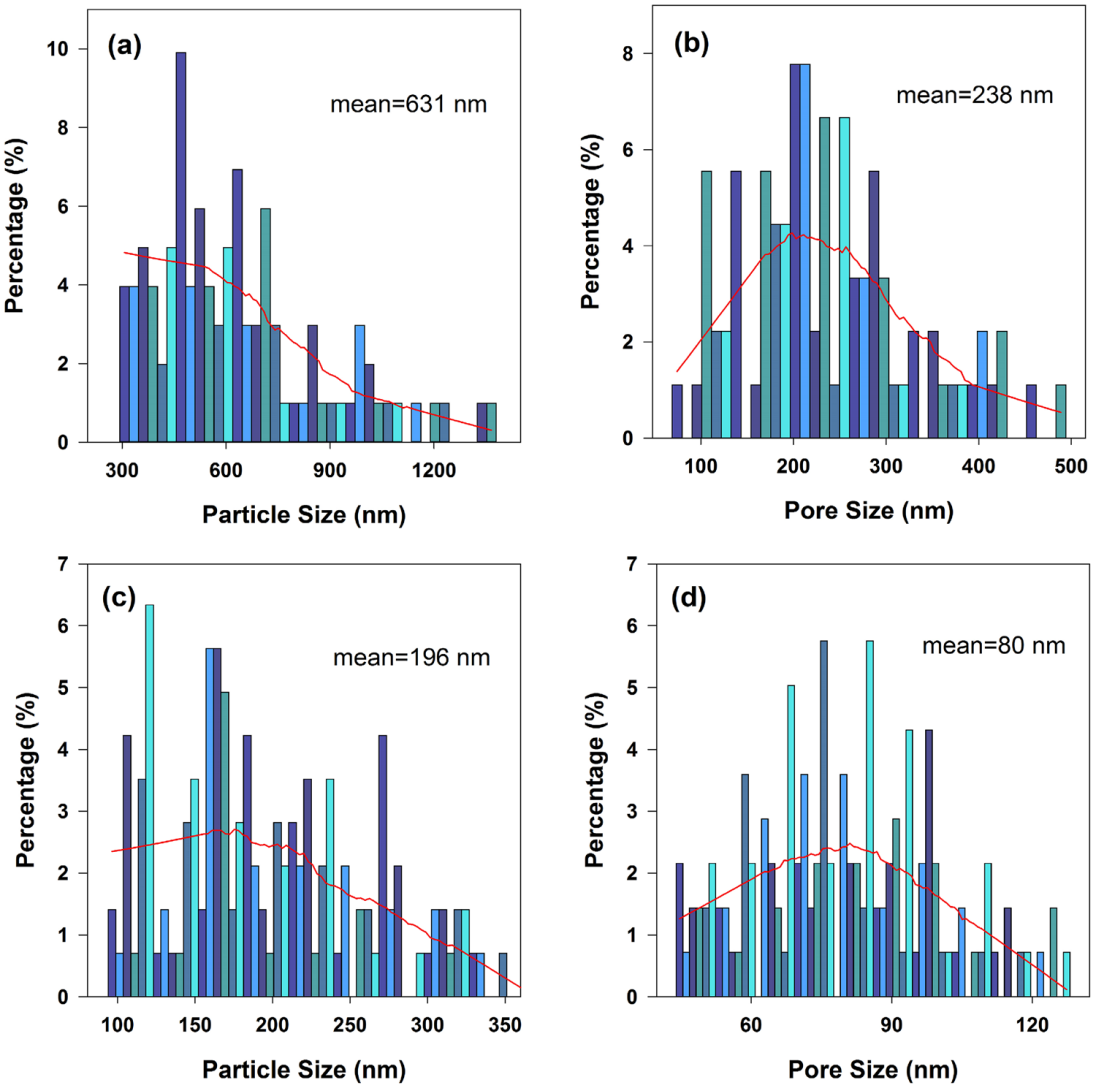
**(a, b)** and **(c, d)** are the granule and nano-pore sizes of the membrane consisting to Fig. 5c and d, respectively.

Figure 6a–d show the granule and nano-pore sizes measured by Image J over 100 sites for each SEM image of Fig. 5c,d. The granule size was taken as the smallest distance of synthesized nano-particles. And similarly, the nano-pore size was estimated by the shortest distance between the adjacent granules. Obviously, the mean nano-particle size is 631 nm for the membrane growing on the surface of Ti particles (Fig. 5c), which is much larger than that of 196 nm on top of the pores of Ti matrix (Fig. 5d). The same rule is obtained for mean nano-pore size, showing considerably different of 238 nm (Fig. 5c) and 80 nm (Fig. 5d), respectively.

It is worth noting that this method of in-situ reaction in confined space is not only potential in fabrication nano-pore membrane as the gradient filtration accuracy layer, but also possibly used in fields of photocatalysis, catalysis after specific modification of membrane. Further research is pursued.

## Conclusion

In summary, Ti–Si intermetallic compound nano-porous membrane was successfully prepared on Ti matrix by an in-situ reactive sintering process. The all synthesized membrane shows the presence of Ti, Ti_5_Si_3_, TiSi and TiO_2_ phases, and the Ti:Si atomic ratio of membrane is about 1.9. Two different synthesized granule configuration zones on membrane are observed. Membrane synthesized on the surface of Ti particles contains the mean sizes of both 631 nm nano-particles and 238 nm nano-pores, which is considerably different from that of membrane growing on top of the micro-pores of Ti matrix, 238 nm nano-particles and 80 nm nano-pores, respectively.
